# A comparison of national cancer registry and direct follow-up in the ascertainment of ovarian cancer

**DOI:** 10.1038/sj.bjc.6690605

**Published:** 1999-08

**Authors:** N MacDonald, K Sibley, A Rosenthal, U Menon, A Jeyarajah, D Oram, I Jacobs

**Affiliations:** Department of Gynaecological Oncology, St Bartholomew's Hospital and The Royal London Hospital School of Medicine and Dentistry, London, EC1A 7BE, UK

**Keywords:** cancer registry, follow-up studies, ovarian cancer

## Abstract

The National Health Service Central Register (NHSCR) and direct follow-up were used to document ovarian and fallopian tube cancers in 22 000 women from 1986 to 1993. Direct follow-up identified 47/49 cases (96%) and the NHSCR 38/49 (78%). NHSCR ascertainment was incomplete and direct follow-up provided additional information. These findings have implications for interpretation of national cancer statistics and for use of the NHSCR in research trials. © 1999 Cancer Research Campaign


*British Journal of Cancer* (1999) **80**(11), 1826–1827
© 1999 Cancer Research Campaign
Article no. bjoc.1999.0605

Short Communication

### A comparison of national cancer registry and direct follow-up in the ascertainment of ovarian cancer

**N MacDonald, K Sibley, A Rosenthal, U Menon, A Jeyarajah, D Oram and I Jacobs**

Department of Gynaecological Oncology, St Bartholomew's Hospital and The Royal London Hospital School of Medicine and Dentistry, London EC1A 7BE, UK

**Summary** The National Health Service Central Register (NHSCR) and direct follow-up were used to document ovarian and fallopian tube cancers in 22 000 women from 1986 to 1993. Direct follow-up identified 47/49 cases (96%) and the NHSCR 38/49 (78%). NHSCR ascertainment was incomplete and direct follow-up provided additional information. These findings have implications for interpretation of national cancer statistics and for use of the NHSCR in research trials.

**Keywords:** cancer registry; follow-up studies; ovarian cancer

The National Health Service Central Register (NHSCR), part of the Office for National Statistics, is used by researchers as a source of follow-up data in prospective trials where cancer incidence information in required. The completeness of cancer registration varies in different regions of England and Wales and is dependent upon cancer site (Villard-Mackintosh et al, 1988; Swerdlow et al, 1993; Warnakulasuriya et al, 1994; Melia et al, 1995). Studies in the north-west region of England and in Manchester have reported registration rates for ovarian cancer of 98.5% and 72% respectively (Nwene and Smith, 1982; Mukherjee et al, 1991). No national studies comparing independent data sources with national cancer registry statistics for ovarian and fallopian tube cancers have been reported. This analysis was performed in order to provide information about the completeness of national registration of ovarian cancer and to compare it with cancer ascertainment through independent sources in a research setting.

### METHODS

The study population consisted of 22 000 post-menopausal women who participated in a study of screening for ovarian cancer which commenced in 1986 (Jacobs et al, 1988, 1993, 1996). Ovarian and fallopian tube cancers were identified directly (by screening and postal questionnaire follow-up) and via the NHSCR. All cases notified from one of these sources were verified by review of histopathology findings.

### Direct follow-up

The research unit documented cases directly through screening and through responses to three annual questionnaires sent to all study participants between 1990 and 1993. The questionnaires requested details of any medical consultations or hospital attendances since registration with the study.

*Received 10 September 1998*
*Revised 11 January 1999*
*Accepted 1 February 1999*

*Correspondence to:* I Jacobs

### NHSCR follow-up

The study cohort was traced through the NHSCR in July 1997 on the basis of name, address and date of birth provided in computerized format. The NHSCR provided cancer registration details for participants who were registered with cancer at any site. In addition, death certificates were provided for participants who had died, whatever the cause of death. Ovarian and fallopian tube cancers ascertained by direct follow-up, but not by the NHSCR in the initial search in July 1997, were resubmitted several times for further searches of all central and regional NHSCR data sources. The final repeat search to locate missed cases took place in May 1998.

In order to allow a comparison of direct and NHSCR follow-up, the analysis was limited to cases of primary epithelial ovarian and fallopian tube cancers (ICD-9 code 183) treated in England and Wales which were diagnosed between 1986 and the last postal questionnaire follow-up in 1993. Restricting the comparison to cases diagnosed in this time period allowed a time lag of more than 4 years after diagnosis for information to reach the NHSCR. Cases of adenocarcinoma of uncertain primary site and of metastatic disease to the ovaries from other primary sites were excluded.

### RESULTS

A total of 49 cases (46 epithelial ovarian and three fallopian tube cancers) were ascertained through all available sources from 1986 to 1993 (Figure 1). The NHSCR identified 38 of these cases (78%) of which 31 were registered and seven were documented by death certification only. Direct follow-up identified 47 cases (96%) of which 19 were detected through screening and 28 by postal questionnaire. The two cases identified by the NHSCR only were not documented directly because the patients did not complete and return postal questionnaires following diagnosis of their cancers. The NHSCR failed to document 11 cases which were identified by direct follow-up. One of these 11 patients was incorrectly registered as having had mediastinal cancer in the year of diagnosis of her histologically confirmed ovarian cancer and another patient was incorrectly registered as having a skin cancer in another year. Two patients were registered by the NHSCR as having ovarian cancer on the basis of death certification, but on histopathological

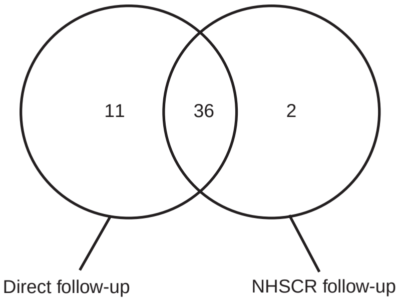

**Figure 1** Summary of case ascertainment of ovarian and fallopian tube cancers in a cohort of 22 000 post-menopausal women from 1986 to 1993 by the National Health Service Central Register (NHSCR) and by direct follow-up review were revealed to have metastatic cancers from the breast and colon to the ovary.

### DISCUSSION

This is the first study to assess NHSCR registration of ovarian and fallopian tube cancers amongst a national cohort of women. The results suggest that NHSCR records are incomplete as 22% of cases were not registered. These findings cannot be explained by incorrect registration under another cancer site as only two of the 11 missed cases were reported by the NHSCR as having cancer of any site. We have excluded the possibility that the ascertainment of ovarian and fallopian tube cancers by independent sources was incorrect by review of histopathological findings in all cases. We also excluded the possibility that the 11 cases were missed because of inaccurate information being provided to the NHSCR, by resubmitting these cases with carefully checked data for further searching. It remains possible that the 11 cases not documented by the NHSCR will eventually be registered and that this study has underestimated the overall accuracy of the NHSCR. However, a lengthy time lag of over 4 years was allowed for the delay in information reaching the NHSCR, and for the purposes of many research trials it is not practicable to wait longer than 4 years for information about cancer registration.

This evidence of incomplete registration of ovarian cancer has several implications. First, it suggests that published national cancer statistics for ovarian cancer may underestimate the true incidence of ovarian cancer, as suggested by previous regional studies (Nwene and Smith, 1982; Mukherjee et al, 1991). It will be important to consider this caveat when using NHSCR data for future epidemiological analyses and to undertake comparisons with other countries. Second, researchers who plan to use NHSCR follow-up in prospective trials will need to be aware of the limitations of this method for documentation of ovarian and fallopian tube cancer. A considerable period of time must be allowed between completion of a study and tracing through the NHSCR to allow data to be registered centrally. Furthermore, it would appear that a complementary method of follow-up is also required. Our analysis provides encouraging evidence for the efficacy of follow-up by postal questionnaire, which also has the benefit of relatively rapid documentation of cancers. Direct follow-up identified 96% of cases and documented 11 cases not reported by the NHSCR. Third, it is possible that the limitations in NHSCR documentation are even greater for some other cancers than for ovarian cancer. The high mortality to incidence ratio of ovarian cancer will often provide an opportunity for ascertainment through death certification even if registration does not occur at the time of diagnosis. This is less frequently the case for cancers with a better prognosis than ovarian cancer.

In summary, this study has identified limitations of NHSCR follow-up in the documentation of ovarian and fallopian tube cancer. In the research context, some of these limitations can be overcome by the use of an independent, direct method of follow-up based on postal questionnaire. Improvements to the organization of cancer registration, including computerization, may improve the situation in due course but at present the limitations of the NHSCR should be recognized.

### ACKNOWLEDGEMENT

The Ovarian Cancer Screening Project, AR and NM were supported by funding from the Charities ROC (Research into Ovarian Cancer) and GCRF (Gynaecology Cancer Research Fund). UM was supported by a Clinical Training Fellowship Grant from the St. Bartholomew’s Hospital Joint Research Board.

### REFERENCES

Jacobs IJ, Stabile I, Bridges J, Kemsley P, Reynolds C, Grudzinskas J and Oram D (1988) Multimodal approach to screening for ovarian cancer. *Lancet* **i**: 268–271

Jacobs IJ, Davies AP, Bridges J, Stabile I, Fay T, Lower A and Grudzinskas JG (1993) Prevalence screening for ovarian cancer in postmenopausal women by CA 125 measurement and ultrasonography. *Br Med J* **306**: 1030–1034

Jacobs IJ, Skates S, Davies AP, Woolas RP, Jeyarajam A, Weidemann P, Sibley K and Oram DH (1996) Risk of diagnosis of ovarian cancer after raised serum CA 125 concentration: a prospective cohort study. *Br Med J* **313**: 1355–1358

Melia J, Frost T, Graham-Brown R, Hunter J, Marsden A, du Vivier A, Warin AP, White J, Whitehead S, Wroughton M, Ellmann R and Chamberlain J (1995) Problems with registration of cutaneous malignant melanoma in England. *Br J Cancer* **72**: 224–228

Mukherjee AK, Leck I, Langley FA and Ashcroft C (1991) The completeness and accuracy of health authority and cancer registry records according to a study of ovarian neoplasms. *Public Health* **105**: 69–78

Nwene U and Smith A (1982) Assessing completeness of cancer registration in the north-western region of England by a method of independent comparison. *Br J Cancer* **46**: 635–639

Swerdlow AJ, Douglas AJ, Vaughan Hudson G and Vaughan Hudson B (1993) Completeness of cancer registration in England and Wales: an assessment based on 2,145 patients with Hodgkin’s disease independently registered by the British National Lymphoma Investigation. *Br J Cancer* **67**: 326–369

Villard-Mackintosh L, Coleman MP and Vessey M (1988) The completeness of cancer registration in England: an assessment from the Oxford-FPA contraceptive study. *Br J Cancer* **58**: 507–511

Warnakulasuriya KA, Acworth P, Bell J and Johnson NW (1994) Incompleteness of oral cancer registration in south-east England. *Br J Cancer* **70**: 736–738

© 1999 Cancer Research Campaign

